# Why did individuals seeking COVID-19 advice return to internet hospitals for follow-up in the early pandemic: Insights from machine learning

**DOI:** 10.1186/s12889-025-25137-2

**Published:** 2025-11-10

**Authors:** Xiaopu Zhang, Qiang Wang, Ling Yang, Tong Su

**Affiliations:** 1 Department of Emergency, The Third People’s Hospital of Changzhou, Changzhou, Jiangsu 213000 China; 2 Department of Cardiology, Changzhou Maternal and Child Health Care Hospital, Changzhou Medical Center, Nanjing Medical University, Changzhou, Jiangsu 213000 China; 3 Department of Cardiology, The Third Affiliated Hospital of Soochow University, Changzhou, Jiangsu 213000 China

**Keywords:** Machine learning, Feature selection, Resampling techniques, Internet hospitals, Consultation on COVID-19, Triage services, Health belief model

## Abstract

**Background:**

Exploring the factors influencing individuals seeking coronavirus disease 2019 (COVID-19) consultation to choose internet hospitals for follow-up care after their initial in-person visit is crucial for optimizing medical resource allocation and enhancing future infectious disease control. This study, anchored in the core constructs of the Health Belief Model, aims to systematically examine the key determinants influencing patients’ decision to pursue a second COVID-19 consultation via an Internet hospital following an initial face-to-face visit.

**Methods:**

This study included 1,055 individuals seeking advice about COVID-19 who consulted an internet hospital during the early outbreak. Model training and evaluation were conducted using stratified five-fold cross‐validation; in each fold, 844 subjects were allocated to the training set and 211 to the test set. We employed a full-feature baseline alongside four feature selection methods—analysis of variance (ANOVA), Boruta, least absolute shrinkage and selection operator (Lasso), and all subsets regression (ASR), and three resampling techniques: Oversampling, Oversampling & Undersampling, and Artificial Synthesis Dataset. Eight machine learning models—logistic regression (LR), support vector machine (SVM), k-nearest neighbors (KNN), random forest (RF), fully connected neural network (FCNN), and extreme gradient boosting (XGBoost), light gradient boosting machine (LightGBM), and categorical boosting (CatBoost)—were constructed to compare performance metrics and rank variable importance in the best-performing model.

**Results:**

The impact of different feature selection methods and resampling techniques on model performance varied. Statistically significant differences were observed in the performance of each model in terms of the area under the receiver operating characteristic curve (AUROC) and the area under the precision-recall curve (AUPRC) (AUROC: *P* < 0.001, AUPRC: *P* < 0.001). Following Boruta, the RF model using the Artificial Synthesis Dataset demonstrated the highest AUROC at 0.946 ± 0.027, whereas the LightGBM model trained on the original imbalanced dataset achieved the highest AUPRC at 0.864 ± 0.070. The key factors influencing the decision to use internet hospitals for follow-up consultations included taking medication on their own or as prescribed by offline doctors before the second visit, chronic respiratory diseases history, contact, consultation purposes, fatigue, cough, and sore throat.

**Conclusions:**

Machine learning may help identify factors influencing the choice of internet hospitals for follow-up consultations. Integrating the Health Belief Model enables a more nuanced understanding of individuals’ online consultation behaviors. Optimizing triage services could enhance the quality and accessibility of internet hospitals, offering valuable insights for future public health events.

## Introduction

Internet hospitals leverage advanced technologies such as the Internet and the Internet of Things, to provide individuals with efficient and convenient telemedicine services, facilitating easier access to medical resources [1–3]. In recent years, China has made significant progress in promoting the establishment of internet hospitals and improving related policies [4]. Particularly, in response to the outbreak of coronavirus disease 2019 (COVID-19), many countries have implemented stringent containment measures [5–7], while simultaneously accelerating the development of telemedicine systems, leading to a substantial increase in the number of consultations conducted via internet hospitals worldwide [8–10].

The advantages of internet hospitals are particularly evident in infectious disease prevention and control. During the early stages of the COVID-19 outbreak, online specialized consultations not only alleviated public anxiety [11], but also enabled the timely identification of high-risk individuals, facilitating epidemiological screening [12]. Additionally, this remote medical model helped reduce cross-infection risks associated with in-person visits [13–15]. However, the development of internet hospitals still faces numerous challenges, such as low adoption rates among elderly individuals, delayed responses from physicians, suboptimal service attitudes, and a mismatch between medical expertise and individuals’ actual needs [16]. These issues undermine individual trust in internet hospitals and underscore the urgent need to optimize telemedicine services. Addressing these challenges necessitates further in-depth research [4].

The Health Belief Model (HBM) serves as a classic framework for elucidating the decision-making processes underlying individual health behaviors [17], and it is widely applied in the field of mobile healthcare [18, 19]. Based on the six constructs of the HBM—perceived susceptibility, perceived severity, perceived barriers, perceived benefits, self-efficacy, and cues to action—we categorized and extracted data features such as individual users’ consultation content and interaction logs on the Internet hospital platform. Analyzing individuals’ willingness to use internet hospitals, particularly the factors influencing their decision to choose internet-based follow-up consultations after an initial in-person visit, can provide a more comprehensive understanding of individual needs. This, in turn, can facilitate improvements in service details and enable the provision of more personalized medical services. The application of machine learning (ML) and deep learning in the medical field has been extensive [20], demonstrating remarkable utility during the COVID-19 pandemic [21–23]. Therefore, This study, integrating the HBM, aims to analyze the different consultation patterns of individuals seeking COVID-19 advice through internet hospitals during the early stages of the pandemic and identify influencing factors using multiple ML algorithms to develop predictive models that support scientific decision-making in internet hospital triage systems.

## Materials and methods

### Data collection

By January 30, 2020, all provinces in China had initiated a Level 1 emergency response to the major public health event. By mid-February, the number of both offline and online consultations related to COVID-19 in Wuhan had begun to decline [15]. This study retrospectively included individuals who consulted the COVID-19 section of the researchers’ affiliated online hospital between January 30 and February 17, 2020. Relevant variables for analysis were extracted in accordance with the definitions provided in Table 1.

### Study definitions

The research objectives were explicitly defined by adhering to the workflow of “individuals providing raw consultation data → physicians making clinical diagnostic decisions”. Individuals were categorized on the basis of their consultation patterns: first attendance (FA): individuals who opted for an online hospital for their initial consultation; second attendance (SA): individuals who initially sought care at an offline hospital but chose an online hospital for their second consultation. Internet telemedicine physicians and fever-clinic physicians independently performed categorical coding of the feature variables corresponding to the six constructs of the HBM (Table 1).

Discrepancies were resolved through discussion among all the research team members. Inter-coder agreement was assessed using Cohen’s Kappa coefficient, which yielded Kappa = 0.82 (>0.75). Individuals who did not meet the inclusion criteria were excluded on the basis of the following criteria: (1) incomplete medical records; (2) consultation for the same purpose at another online hospital prior to this visit; and (3) consultation unrelated to COVID-19.

**Table 1:** Health belief model constructs operationalization for COVID-19 online consultation Behavior​

HBM constructs and operationalization	Characteristic Variables	Individual Grouping and Definition	Examples
Perceived Susceptibility	Contact	Direct contact: Contact with residents in Hubei Province. Indirect contact: Interaction with individuals returning from Hubei Province.	Direct contact: Cohabitation with family/friends in Wuhan. Indirect contact: Contact with colleagues returning from Hubei.
	Travel	Having a history of domestic travel (outside Hubei Province) or international travel.	Traveled to Beijing for a visit in the past week.
	Crowd	Based on whether one has visited crowded places such as supermarkets, public transportation, and shopping malls.	Commutes to work by subway.
	Current Smoking	Currently smoking.	Smokes half a pack of cigarettes daily.
	Gender	Women are more sensitive to the risk of respiratory infections.	Female clients automatically express anxiety during consultation.
Perceived Severity	Age (years)	Elderly: ≥60 years old.	In their 60 s with underlying diseases.
	chronic respiratory diseases (CRD) history	CRD history is defined as having been diagnosed with chronic obstructive pulmonary disease (COPD), bronchiectasis, emphysema, or asthma in a tertiary hospital.	History of COPD for 5 years.
	Suspected COVID-19 symptoms [24]	Fever: Self-measured oral temperature exceeding 37.3 °C, or axillary temperature exceeding 37.0 °C. Sore Throat, Cough. Chills, Headache, Chest Tightness, Shortness of Breath.	Persistent high fever for 3 days. Sore throat as if cut by a knife. Needs to gasp for breath during activity.
	Pregnant	Current gestation.	10 weeks pregnant with cough.
	Province	To assess the severity of the outbreak in different regions, as of February 18, 2020, the confirmed case reports published by the National Health Commission of China were used as a classification standard: mild: 1–99 cases; mild to moderate: 100–499 cases; moderate: 500–999 cases; moderate to severe: 1,000–9,999 cases; and severe: ≥10,000 cases.	Living in Wuhan.
Perceived Benefits	Problem (Consultation purposes)	Category 1: Individuals with typical or atypical COVID-19 symptoms [24], inquiring about potential COVID-19 infection; Category 2: Asymptomatic individuals, inquiring about the possibility of asymptomatic COVID-19 infection; Category 3: Individuals with common respiratory infection symptoms, inquiring about medication safety and existing treatment options; Category 4: Asymptomatic individuals, inquiring about the transmission routes of COVID-19.	Category 1:Whether the patient is currently infected with COVID-19 if he or she has one or more of the following symptoms, such as high self-measured temperature, symptoms of sore throat and cough, nausea and vomiting, self-consciousness of facial burning, and intermittent feeling of tightness in the chest and dyspnoea. Category 2: I have been in contact with family members, colleagues or friends who have returned from Wuhan or from other infected areas who are currently asymptomatic or have cold symptoms, and I am currently asymptomatic, but whether I have been infected with COVID-19. Category 3: Are Chinese medicines effective in the treatment of COVID-19? I have been taking them for a few days now and there is no improvement in my symptoms, should I continue to take them or are there better medicines that can help? Can I take certain medicines during pregnancy? Category 4: Is the faeces of a COVID-19 patient potentially infectious? does courier delivery pass the COVID-19 virus; If I need to go out, what protective measures do I need to take to prevent the transmission of COVID-19?
	Infection transmission concern	Individuals are concerned that seeking medical treatment in offline hospitals may increase the risk of cross-infection.	“Fear of infection”, “Avoid contact”.
Perceived barriers	Age (years)	Elderly: ≥60 years old.	Cannot operate the inquiry - based app independently.
Cues to Action	Drugs	Took medication on their own or as prescribed by offline doctors before the second visit.	Still has a fever after taking traditional Chinese medicine by oneself.
	Fatigue	Fatigue lasting more than 7 days.	Have been feeling fatigued for more than a week.
Self-efficacy	Age (years)	Young adults: 21–30 years old and 31–40 years old.	Can skillfully upload chest CT reports.
	Consultation made on behalf of another individual	Refers to consultation initiated on behalf of the individual by family members or relatives and friends.	My father has a fever. What medicines does he need to take?

The statistical analysis of epidemiological information started in December 2019 and was defined as the contact history within 14 days prior to the onset of illness [25]

### Statistical analysis

**Fig. 1:**
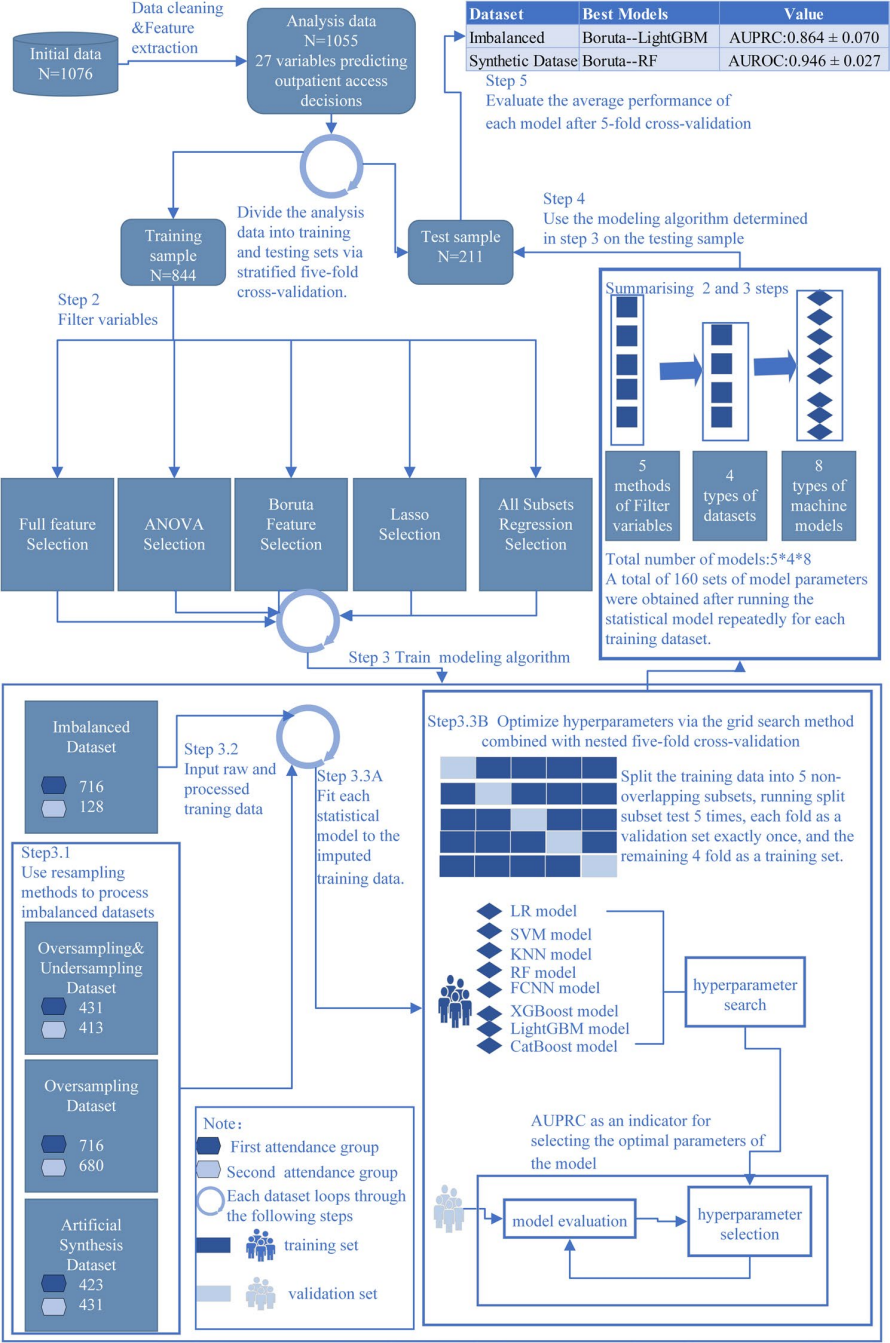
Workflow for creating machine learning models from datasets

This study employed stratified five-fold cross‐validation to partition the dataset, with each fold preserving the proportion of SA. This approach divides the data into training and test sets within each fold, balancing class distributions and minimizing bias from a single train–test split. To maintain reproducibility, the random number of seeds was set to 123. Feature selection was performed via five methods: a full‐feature (Full) baseline and analysis of variance (ANOVA), Boruta, least absolute shrinkage and selection operator (Lasso), and all subset regression (ASR). The training dataset underwent resampling via three techniques: Oversampling, Oversampling & Undersampling, and Artificial Synthesis Dataset. These methods were used to generate balanced datasets with equal proportions of positive and negative samples. ML models were developed via the following algorithms: logistic regression (LR), support vector machine (SVM), k-nearest neighbors (KNN), random forest (RF), and extreme gradient boosting (XGBoost), light gradient boosting machine (LightGBM), and categorical boosting (CatBoost). Hyperparameter optimization was performed via grid search combined with nested five‐fold cross‐validation, using the area under the precision-recall curve (AUPRC) as the primary metric for selecting optimal hyperparameters. Using the test dataset and the optimal hyperparameters, classification models were constructed and evaluated based on the following performance metrics: accuracy, F1 score, matthews correlation coefficient (MCC), area under the receiver operating characteristic curve (AUROC) and AUPRC. The optimal model was selected based on the mean performance of each metric across the five test folds, using a dual‐criteria approach: for the resampled balanced datasets, the model with the highest AUROC value was chosen, whereas for the imbalanced dataset, the model with the highest AUPRC value was selected. For the two selected models, we generated predictions on each test fold using their respective optimal hyperparameters. We then aggregated these predictions to plot receiver operating characteristic curves, precision-recall (PR) curves, and calibration curves, thereby visually assessing each model’s discriminative performance and calibration. Finally, the hyperparameter set that achieved the highest single‐fold AUROC/AUPRC in the chosen model was used to retrain on the entire dataset, and feature importance rankings were computed from this fully retrained model. All the statistical analyses and model development were conducted via R 4.2.1 (R Foundation for Statistical Computing, Vienna, Austria) and open-source R packages, including “createDataPartition”, “GoodmanKruskal”, “rms”, “bestglm”, “randomForest”, “MASS”, “kknn”, “neuralnet”, “keras”, “tensorflow”, “xgboost”, “lightgbm”, “catboost” “SHAPforxgboost”, and “PMCMRplus”. All hypothesis tests were two-tailed, with a significance threshold of *P* < 0.05. Figure 1 shows the workflow for creating ML models from datasets.

## Results

### Online consultation information

A total of 1,055 individuals seeking COVID-19 consultation were included in this study, with a mean age of 35.028 ± 12.801 years. The age distribution differed significantly between the FA and the SA. Elderly individuals were more prevalent in the SA (10.6% vs. 3.35%), whereas younger individuals were more likely to belong to the FA (34.3% vs. 19.4%). There was no significant correlation between SA and the severity of the pandemic in the individuals’ province. The primary reason for first-time online consultations was to assess the likelihood of contracting COVID-19, whereas second-time consultations focused more on medication-related inquiries. Additional factors potentially associated with SA included pregnancy, chronic respiratory diseases (CRD) history, contact, cough, sore throat, nasal congestion, fatigue, and medication usage during the current illness (variable: Drugs). The specific distributions of these characteristics are provided in Table 2.

**Table 2:** Characteristics of the population in the two outpatient attendance patterns

Variable	Overall (*N* = 1055), *n* (%)	FA (*N* = 895), *n* (%)	SA (*N* = 160), *n* (%)	*P* value
Demographic Characteristics				
Age (years):				<0.001
<=20	90 (8.53)	80 (8.94)	10 (6.25)	
21 < = 30	338 (32.0)	307 (34.3)	31 (19.4)	
31 < = 40	346 (32.8)	289 (32.3)	57 (35.6)	
41 < = 50	149 (14.1)	125 (14.0)	24 (15.0)	
51 < = 60	85 (8.06)	64 (7.15)	21 (13.1)	
>=60	47 (4.45)	30 (3.35)	17 (10.6)	
Gender: Male	497 (47.1)	417 (46.6)	80 (50.0)	0.478
Pregnant	16 (1.52)	9 (1.01)	7 (4.38)	0.006
Province:				0.086
Mild	65 (6.16)	59 (6.59)	6 (3.75)	
Mild to Moderate	308 (29.2)	261 (29.2)	47 (29.4)	
Moderate	352 (33.4)	308 (34.4)	44 (27.5)	
Moderate to Severe	241 (22.8)	197 (22.0)	44 (27.5)	
Severe	89 (8.44)	70 (7.82)	19 (11.9)	
Current Smoking	14 (1.33)	12 (1.34)	2 (1.25)	0.999
CRD history	64 (6.07)	10 (1.12)	54 (33.8)	<0.001
Symptom Characteristics				
Cough	306 (29.0)	221 (24.7)	85 (53.1)	<0.001
Fever	266 (25.2)	223 (24.9)	43 (26.9)	0.67
Sore Throat	209 (19.8)	135 (15.1)	74 (46.2)	<0.001
Nasal Congestion	155 (14.7)	99 (11.1)	56 (35.0)	<0.001
Fatigue	122 (11.6)	53 (5.92)	69 (43.1)	<0.001
Chest Tightness	98 (9.29)	88 (9.83)	10 (6.25)	0.197
Expectoration	78 (7.39)	67 (7.49)	11 (6.88)	0.914
Headache	62 (5.88)	58 (6.48)	4 (2.50)	0.074
Diarrhea	45 (4.27)	43 (4.80)	2 (1.25)	0.066
Myalgia	28 (2.65)	24 (2.68)	4 (2.50)	0.999
Shortness of Breath	25 (2.37)	22 (2.46)	3 (1.88)	0.999
Nausea and Vomiting	24 (2.27)	21 (2.35)	3 (1.88)	0.999
Chills	13 (1.23)	11 (1.23)	2 (1.25)	0.999
Conjunctivitis	4 (0.38)	4 (0.45)	0 (0.00)	0.999
Consultation Details & Health Behavior				
Problem:				<0.001
One	772 (73.2)	702 (78.4)	70 (43.8)	
Two	131 (12.4)	128 (14.3)	3 (1.88)	
Three	120 (11.4)	38 (4.25)	82 (51.2)	
Four	32 (3.03)	27 (3.02)	5 (3.12)	
Consultation made on behalf of another individual	26 (2.46)	23 (2.57)	3 (1.88)	0.785
Infection transmission concern	37 (3.51)	33 (3.69)	4 (2.50)	0.604
Drugs	70 (6.64)	5 (0.56)	65 (40.6)	<0.001
Contact:				<0.001
Direct Contact	129 (12.2)	75 (8.38)	54 (33.8)	
Indirect Contact	36 (3.41)	13 (1.45)	23 (14.4)	
Travel	47 (4.45)	44 (4.92)	3 (1.88)	0.131
Crowd	45 (4.27)	41 (4.58)	4 (2.50)	0.323

FA individuals who opted for an online hospital for their initial consultation; SA individuals who initially sought care at an offline hospital but chose an online hospital for their second consultation; CRD history refers to a history of chronic respiratory diseases; Drugs refers to medication usage during the current illness; Problem refers to the purpose of the internet consultation during this visit. Bold text indicates variables with statistical significance

###  Model performance SA

**Fig. 2:**
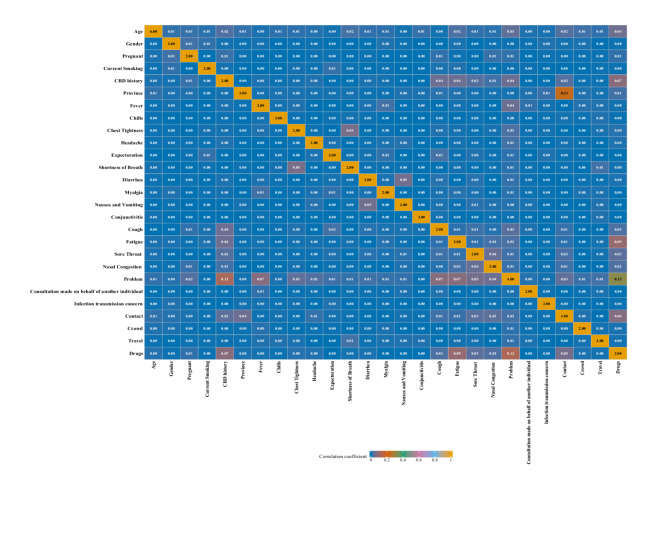
Correlation analysis among independent variables. Note: Correlation analysis among the independent variables. The color gradient represents the magnitude of correlation coefficients, ranging sequentially from 0 (blue), 0.2 (orange-red), 0.4 (cyan-green), 0.6 (purplish-red), 0.8 (light blue) to 1 (orange-yellow). CRD history refers to a history of chronic respiratory diseases; Drugs refers to medication usage during the current illness; Problem refers to the purpose of the internet consultation during this visit

No significant correlations were observed among the independent variables (Fig. 2). The classification accuracy of all models on the test set was excellent (minimum = 0.85), and the effect of data resampling on model performance varied across feature selection methods (Table 3). After applying oversampling, both the Boruta-RF and Boruta-LightGBM models showed increases in accuracy, F1 score, and MCC. In contrast, logistic regression models built on the three resampled datasets exhibited no appreciable change in F1 performance. Similarly, SVM models trained on the original imbalanced dataset (Full-Imbalanced-SVM, ANOVA-Imbalanced-SVM, Boruta-Imbalanced-SVM) achieved the highest MCC. These imbalanced-data SVM also significantly outperformed KNN models under all resampling schemes (Artificial Synthesis, Oversampling & Undersampling, Oversampling, Imbalanced), for example: Full-Imbalanced-SVM versus Full-Artificial Synthesis-KNN (*P* < 0.001) and Boruta-Imbalanced-SVM versus ANOVA- Oversampling & Undersampling-KNN (*P* = 0.039).

**Table 3:** Model performance metrics

Model	Imbalanced			Oversampling Dataset			Oversampling & Undersampling Dataset			Artificial Synthesis Dataset		
	Accuracy	F1 score	MCC	Accuracy	F1 score	MCC	Accuracy	F1 score	MCC	Accuracy	F1 score	MCC
Full												
CatBoost	0.927 ± 0.013	0.958 ± 0.008	0.698 ± 0.050	0.928 ± 0.036	0.958 ± 0.021	0.712 ± 0.141	0.913 ± 0. 425	0.948 ± 0.016	0.673 ± 0.098	0.897 ± 0.029	0.939 ± 0.018	0.628 ± 0.094
FCNN	0.878 ± 0.080	0.871 ± 0.175	0.567 ± 0.188	0.845 ± 0.154	0.849 ± 0.225	0.565 ± 0.168	0.845 ± 0.145	0.856 ± 0.205	0.590 ± 0.127	0.858 ± 0.136	0.858 ± 0.211	0.592 ± 0.185
KNN	0.899 ± 0.010	0.944 ± 0.005	0.547 ± 0.058	0.868 ± 0.027	0.923 ± 0.017	0.465 ± 0.075	0.813 ± 0.023	0.886 ± 0.015	0.374 ± 0.078	0.831 ± 0.046	0.899 ± 0.030	0.386 ± 0.099
LR	0.933 ± 0.020	0.962 ± 0.011	0.718 ± 0.088	0.905 ± 0.014	0.943 ± 0.008	0.661 ± 0.063	0.904 ± 0.018	0.942 ± 0.011	0.668 ± 0.066	0.918 ± 0.029	0.952 ± 0.018	0.697 ± 0.092
LightGBM	0.926 ± 0.023	0.957 ± 0.014	0.713 ± 0.086	0.919 ± 0.022	0.952 ± 0.014	0.706 ± 0.081	0.906 ± 0.023	0.945 ± 0.015	0.675 ± 0.082	0.910 ± 0.020	0.946 ± 0.012	0.681 ± 0.078
RF	0.870 ± 0.025	0.929 ± 0.013	0.318 ± 0.180	0.934 ± 0.015	0.962 ± 0.008	0.727 ± 0.067	0.908 ± 0.014	0.945 ± 0.008	0.656 ± 0.057	0.910 ± 0.016	0.947 ± 0.009	0.661 ± 0.074
SVM	0.940 ± 0.024	0.966 ± 0.014	0.749 ± 0.108	0.924 ± 0.023	0.956 ± 0.012	0.724 ± 0.087	0.905 ± 0.022	0.943 ± 0.013	0.671 ± 0.082	0.922 ± 0.021	0.953 ± 0.013	0.692 ± 0.084
XGBoost	0.927 ± 0.020	0.960 ± 0.011	0.690 ± 0.091	0.897 ± 0.017	0.938 ± 0.011	0.632 ± 0.050	0.875 ± 0.043	0.923 ± 0.028	0.599 ± 0.098	0.892 ± 0.021	0.935 ± 0.014	0.627 ± 0.059
ANOVA												
CatBoost	0.928 ± 0.021	0.959 ± 0.012	0.697 ± 0.094	0.913 ± 0.015	0.948 ± 0.009	0.675 ± 0.062	0.887 ± 0.032	0.932 ± 0.020	0.598 ± 0.101	0.881 ± 0.040	0.927 ± 0.026	0.600 ± 0.094
FCNN	0.852 ± 0.114	0.873 ± 0.174	0.663 ± 0.086	0.867 ± 0.106	0.853 ± 0.216	0.585 ± 0.136	0.868 ± 0.064	0.839 ± 0.224	0.558 ± 0.110	0.825 ± 0.196	0.817 ± 0.295	0.574 ± 0.176
KNN	0.913 ± 0.016	0.951 ± 0.008	0.623 ± 0.092	0.888 ± 0.032	0.934 ± 0.020	0.566 ± 0.112	0.873 ± 0.028	0.924 ± 0.017	0.536 ± 0.079	0.876 ± 0.016	0.926 ± 0.010	0.549 ± 0.071
LR	0.930 ± 0.026	0.960 ± 0.014	0.698 ± 0.124	0.905 ± 0.029	0.943 ± 0.018	0.666 ± 0.085	0.910 ± 0.016	0.946 ± 0.010	0.682 ± 0.041	0.911 ± 0.027	0.946 ± 0.017	0.688 ± 0.088
LightGBM	0.927 ± 0.030	0.957 ± 0.018	0.719 ± 0.115	0.906 ± 0.031	0.943 ± 0.019	0.672 ± 0.101	0.897 ± 0.028	0.938 ± 0.018	0.646 ± 0.084	0.907 ± 0.021	0.944 ± 0.013	0.670 ± 0.085
RF	0.903 ± 0.019	0.946 ± 0.010	0.566 ± 0.106	0.915 ± 0.030	0.945 ± 0.014	0.680 ± 0.100	0.900 ± 0.020	0.940 ± 0.013	0.643 ± 0.069	0.898 ± 0.026	0.939 ± 0.016	0.637 ± 0.091
SVM	0.937 ± 0.025	0.964 ± 0.014	0.739 ± 0.107	0.914 ± 0.023	0.949 ± 0.014	0.677 ± 0.086	0.898 ± 0.018	0.939 ± 0.012	0.638 ± 0.054	0.899 ± 0.004	0.939 ± 0.003	0.648 ± 0.030
XGBoost	0.927 ± 0.020	0.958 ± 0.011	0.690 ± 0.091	0.892 ± 0.047	0.935 ± 0.030	0.628 ± 0.111	0.873 ± 0.039	0.922 ± 0.026	0.602 ± 0.092	0.880 ± 0.038	0.927 ± 0.025	0.614 ± 0.090
Boruta												
CatBoost	0.932 ± 0.026	0.961 ± 0.015	0.709 ± 0.118	0.914 ± 0.019	0.948 ± 0.012	0.677 ± 0.075	0.880 ± 0.046	0.926 ± 0.029	0.610 ± 0.103	0.900 ± 0.015	0.940 ± 0.010	0.655 ± 0.052
FCNN	0.843 ± 0.125	0.738 ± 0.336	0.671 ± 0.083	0.867 ± 0.125	0.913 ± 0.094	0.629 ± 0.144	0.838 ± 0.179	0.871 ± 0.181	0.603 ± 0.168	0.850 ± 0.139	0.868 ± 0.178	0.600 ± 0.140
KNN	0.912 ± 0.009	0.951 ± 0.005	0.614 ± 0.047	0.896 ± 0.030	0.938 ± 0.019	0.617 ± 0.081	0.896 ± 0.025	0.938 ± 0.016	0.619 ± 0.065	0.891 ± 0.037	0.934 ± 0.025	0.620 ± 0.079
LR	0.917 ± 0.027	0.953 ± 0.014	0.631 ± 0.135	0.906 ± 0.020	0.944 ± 0.012	0.672 ± 0.061	0.913 ± 0.021	0.947 ± 0.013	0.702 ± 0.072	0.911 ± 0.017	0.947 ± 0.010	0.691 ± 0.070
LightGBM	0.872 ± 0.023	0.927 ± 0.009	0.633 ± 0.047	0.915 ± 0.019	0.949 ± 0.012	0.694 ± 0.072	0.906 ± 0.018	0.944 ± 0.011	0.670 ± 0.074	0.903 ± 0.020	0.942 ± 0.012	0.664 ± 0.069
RF	0.906 ± 0.014	0.948 ± 0.008	0.583 ± 0.077	0.920 ± 0.015	0.953 ± 0.009	0.701 ± 0.057	0.910 ± 0.011	0.946 ± 0.007	0.677 ± 0.028	0.917 ± 0.014	0.950 ± 0.008	0.697 ± 0.061
SVM	0.937 ± 0.027	0.964 ± 0.016	0.737 ± 0.117	0.910 ± 0.012	0.946 ± 0.007	0.671 ± 0.061	0.909 ± 0.017	0.945 ± 0.010	0.675 ± 0.064	0.909 ± 0.010	0.946 ± 0.007	0.670 ± 0.046
XGBoost	0.926 ± 0.021	0.957 ± 0.012	0.685 ± 0.095	0.876 ± 0.042	0.926 ± 0.029	0.597 ± 0.087	0.869 ± 0.040	0.920 ± 0.027	0.586 ± 0.087	0.875 ± 0.034	0.923 ± 0.023	0.600 ± 0.072
Lasso												
CatBoost	0.926 ± 0.027	0.958 ± 0.015	0.683 ± 0.124	0.888 ± 0.049	0.932 ± 0.032	0.625 ± 0.125	0.884 ± 0.035	0.930 ± 0.023	0.618 ± 0.088	0.888 ± 0.047	0.932 ± 0.031	0.612 ± 0.120
FCNN	0.891 ± 0.063	0.846 ± 0.200	0.661 ± 0.084	0.880 ± 0.075	0.913 ± 0.082	0.633 ± 0.079	0.888 ± 0.056	0.934 ± 0.033	0.644 ± 0.045	0.880 ± 0.070	0.919 ± 0.064	0.597 ± 0.119
KNN	0.914 ± 0.024	0.951 ± 0.013	0.626 ± 0.114	0.885 ± 0.045	0.931 ± 0.029	0.603 ± 0.125	0.881 ± 0.047	0.928 ± 0.030	0.589 ± 0.130	0.869 ± 0.047	0.920 ± 0.030	0.563 ± 0.127
LR	0.921 ± 0.026	0.956 ± 0.014	0.657 ± 0.123	0.913 ± 0.037	0.948 ± 0.023	0.673 ± 0.126	0.909 ± 0.037	0.946 ± 0.023	0.662 ± 0.126	0.916 ± 0.035	0.950 ± 0.022	0.683 ± 0.126
LightGBM	0.895 ± 0.020	0.937 ± 0.013	0.635 ± 0.067	0.919 ± 0.027	0.952 ± 0.016	0.696 ± 0.101	0.886 ± 0.068	0.930 ± 0.043	0.642 ± 0.181	0.853 ± 0.036	0.908 ± 0.024	0.574 ± 0.090
RF	0.904 ± 0.024	0.945 ± 0.016	0.613 ± 0.061	0.906 ± 0.029	0.944 ± 0.017	0.664 ± 0.106	0.913 ± 0.043	0.948 ± 0.026	0.677 ± 0.141	0.896 ± 0.062	0.937 ± 0.039	0.634 ± 0.184
SVM	0.931 ± 0.026	0.960 ± 0.015	0.707 ± 0.117	0.892 ± 0.023	0.935 ± 0.015	0.617 ± 0.093	0.894 ± 0.028	0.936 ± 0.017	0.629 ± 0.104	0.891 ± 0.043	0.934 ± 0.027	0.635 ± 0.129
XGBoost	0.925 ± 0.026	0.957 ± 0.015	0.675 ± 0.123	0.876 ± 0.040	0.924 ± 0.027	0.595 ± 0.091	0.871 ± 0.037	0.921 ± 0.025	0.590 ± 0.085	0.876 ± 0.047	0.922 ± 0.029	0.585 ± 0.136
ASR												
CatBoost	0.928 ± 0.024	0.959 ± 0.013	0.690 ± 0.114	0.906 ± 0.034	0.943 ± 0.021	0.655 ± 0.126	0.887 ± 0.033	0.932 ± 0.021	0.619 ± 0.086	0.889 ± 0.025	0.933 ± 0.016	0.619 ± 0.097
FCNN	0.889 ± 0.049	0.913 ± 0.056	0.580 ± 0.108	0.878 ± 0.055	0.927 ± 0.034	0.604 ± 0.078	0.888 ± 0.029	0.931 ± 0.024	0.601 ± 0.055	0.897 ± 0.020	0.931 ± 0.029	0.636 ± 0.059
KNN	0.921 ± 0.022	0.958 ± 0.013	0.657 ± 0.109	0.901 ± 0.030	0.942 ± 0.018	0.623 ± 0.117	0.897 ± 0.029	0.939 ± 0.017	0.614 ± 0.101	0.874 ± 0.041	0.923 ± 0.026	0.580 ± 0.121
LR	0.917 ± 0.019	0.953 ± 0.010	0.634 ± 0.097	0.901 ± 0.040	0.941 ± 0.025	0.645 ± 0.129	0.897 ± 0.028	0.938 ± 0.017	0.633 ± 0.085	0.904 ± 0.028	0.943 ± 0.017	0.658 ± 0.102
LightGBM	0.915 ± 0.017	0.950 ± 0.010	0.674 ± 0.090	0.908 ± 0.027	0.945 ± 0.016	0.669 ± 0.098	0.901 ± 0.028	0.940 ± 0.017	0.654 ± 0.091	0.889 ± 0.025	0.933 ± 0.015	0.630 ± 0.094
RF	0.920 ± 0.028	0.955 ± 0.015	0.654 ± 0.128	0.910 ± 0.031	0.947 ± 0.018	0.657 ± 0.115	0.900 ± 0.038	0.940 ± 0.023	0.643 ± 0.123	0.892 ± 0.027	0.935 ± 0.016	0.620 ± 0.109
SVM	0.931 ± 0.030	0.960 ± 0.017	0.705 ± 0.135	0.900 ± 0.021	0.940 ± 0.013	0.628 ± 0.095	0.896 ± 0.025	0.938 ± 0.015	0.627 ± 0.094	0.871 ± 0.041	0.920 ± 0.026	0.599 ± 0.111
XGBoost	0.922 ± 0.024	0.956 ± 0.013	0.665 ± 0.112	0.891 ± 0.046	0.934 ± 0.030	0.628 ± 0.102	0.874 ± 0.042	0.923 ± 0.028	0.599 ± 0.078	0.871 ± 0.021	0.922 ± 0.012	0.573 ± 0.094

*Full* full-features, *ANOVA* analysis of variance, *Boruta* Boruta feature selection, *Lasso* least absolute shrinkage and selection operator, *ASR* all subset regression, *MCC* matthews correlation coefficient, *LR* logistic regression, *SVM* support vector machine, *KNN* k-nearest neighbors, *RF* random forest, *FCNN* fully connected neural network, *XGBoost* extreme gradient boosting, *LightGBM* light gradient boosting machine, *CatBoost* categorical boosting

**Fig. 3:**
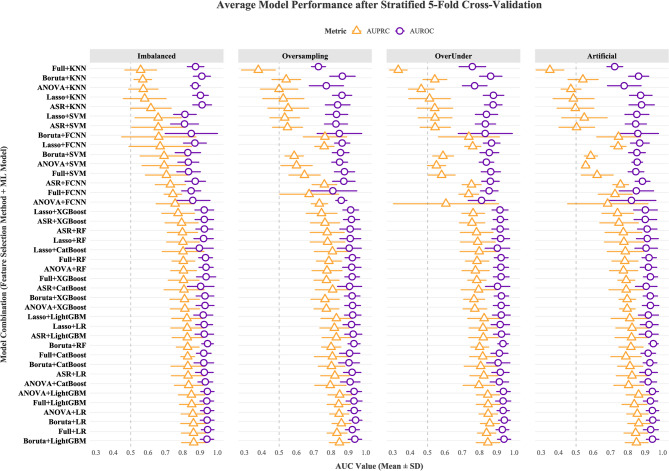
Benchmarking machine learning workflows via stratified 5-fold cross-validation: Stratified point-range plots of AUROC and AUPRC (mean ± SD) with a faceted design. Note: The horizontal bar chart presents the mean AUC values (±1 standard deviation) of 40 model combinations, categorized by four data balancing strategies (from left to right: Imbalanced, Oversampling, OverUnder, and Artificial Synthesis). Each combination integrates feature selection methods (denoted as prefixes) and machine learning algorithms (denoted as suffixes). Two metrics are displayed in the chart: purple circles represent the mean AUROC values, and yellow triangles represent the mean AUPRC values. The y-axis lists model workflows grouped by balancing methods, while the x-axis indicates the range of AUC values (ranging from 0.3 to 1.0). Imbalanced: Original imbalanced dataset; OverUnder: Oversampling & Undersampling dataset; Artificial: Artificial Synthesis dataset; Full: full-features; ANOVA: analysis of variance; Boruta: Boruta feature selection; Lasso: least absolute shrinkage and selection operator; ASR: All Subsets Regression. LR: Logistic Regression; SVM: Support Vector Machine; kNN: k-Nearest Neighbor; RF: Random Forest; FCNN: Fully Connected Neural Network; XGBoost: eXtreme Gradient Boosting; LightGBM: Light Gradient Boosting Machine; CatBoost: Categorical Boosting; AUROC: area under the receiver operating characteristic curve; AUPRC: area under the precision-recall curve

Figure 3 presents the mean AUROC and AUPRC for each model in classifying SA on the test set. The Friedman test revealed statistically significant differences among the models for both metrics (AUROC: *P* < 0.001; AUPRC: *P* < 0.001). Post hoc pairwise comparisons were conducted with independent two-sample z‐tests, and *P*‐values were adjusted for multiple testing using the Benjamini–Hochberg procedure. In terms of AUPRC, the LightGBM model built with Boruta demonstrated the best performance on the imbalanced dataset. Its performance was not statistically different from that of LR, CatBoost, or XGBoost models built under the same configuration (vs. LR: *P* = 0.965; vs. CatBoost: *P* = 0.618; vs. XGBoost: *P* = 0.417), but it was significantly superior to SVM, KNN, and FCNN models employing alternative feature selection and resampling strategies (e.g., vs. ANOVA-Artificial Synthesis-KNN: *P* < 0.001; vs. ANOVA-Oversampling-FCNN: *P* = 0.002; vs. ASR-Artificial Synthesis-FCNN: *P* = 0.016; vs. Boruta-Imbalanced-SVM: *P* = 0.043; vs. Full-*Oversampling & Undersampling*-KNN: *P* < 0.001). In terms of AUROC, the Artificial Synthesis-Boruta-RF model showed no statistically significant difference in performance compared with the Boruta-LR model (*P* = 0.999); however, its performance remained among the highest of all models and was significantly superior to SVM and KNN models across multiple configurations (e.g., ANOVA-Artificial Synthesis-SVM: *P* < 0.001; ASR-*Oversampling & Undersampling*-SVM: *P* = 0.001; Full-Artificial Synthesis-KNN: *P* < 0.001).

To evaluate the benefit of feature selection methods, the present study used the Full model, incorporating all predictors, as the baseline for comprehensive comparison. Statistical analyses showed that, in the LightGBM model constructed on the imbalanced dataset, the Boruta method demonstrated no statistically significant differences from the Full method in MCC, AUPRC, or AUROC (MCC: *P* = 0.272; AUPRC: *P* = 0.999; AUROC: *P* = 0.999). Similarly, in the RF model built on the Artificial Synthesis dataset, the Full method did not exhibit significantly superior performance over models using other feature selection methods across all evaluation metrics (*P* > 0.05 for all comparisons), with the smallest *P*-value observed in the comparison between the ASR and Full models for the F1 metric (*P* = 0.144).

**Fig. 4:**
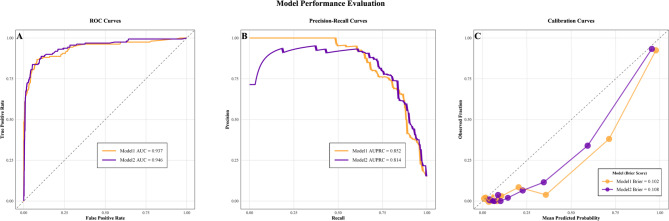
Performance evaluation of dual-dataset dual-criteria optimized models using stratified 5-fold cross-validation. Note: (**A**) Discrimination performance illustrated by Receiver Operating Characteristic (ROC) curves, with the AUROC quantified. (**B**) Precision-recall trade-off, critical for imbalanced datasets, illustrated by Precision-Recall curves, with the AUPRC quantified. (**C**) Calibration (Calibration Curve): Predicted probability (x-axis) versus observed event frequency (y-axis). Marker size denotes sample count per probability interval (*n* = 5 bins). Model 1: Imbalanced-Boruta-LightGBM model. Model 2: Artificial-Boruta-RF model. Imbalanced: Original imbalanced dataset; Artificial: Artificial Synthesis dataset; Boruta: Boruta feature selection; RF: Random Forest; LightGBM: Light Gradient Boosting Machine; AUROC: area under the receiver operating characteristic curve; AUPRC: area under the precision-recall curve

Figure 4 displays the ROC, PR, and calibration curves for the two top-performing models identified in Fig. 3. Both Model 1 and Model 2 exhibited excellent discriminative ability, with Model 2 showing a slight edge across all classification thresholds (AUROC = 0.946 vs. 0.937; ΔAUROC = 0.009; Delong test *P* = 0.412), while Model 1 achieved a superior PR trade‐off (AUPRC = 0.852 vs. 0.814 for Model 2; permutation test *P* = 0.361). Moreover, Model 1 demonstrated better probability calibration (Brier score = 0.102 vs. 0.108 for Model 2), although both models attained satisfactory calibration.

### Significant features

**Fig. 5:**
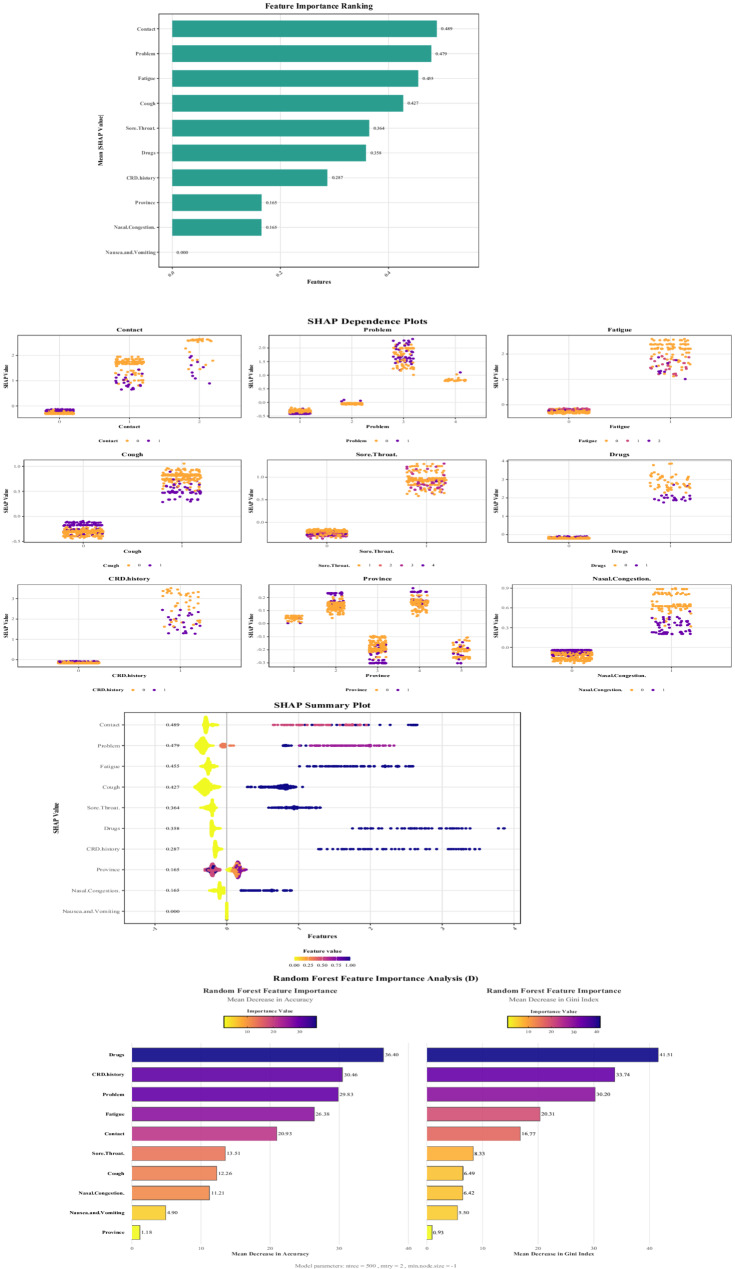
**A**. LightGBM feature importance ranking. Note: X-axis: Mean absolute SHAP value (Mean |SHAP value|), quantifying the average magnitude of feature influence on model output. Higher values indicate greater feature contribution to model decisions. Y-axis: Input features ranked in descending order by mean absolute SHAP value. Bars: Turquoise-colored bars represent the mean absolute SHAP value for each feature. Numeric annotations: Values to the right of features denote the precise mean absolute SHAP value. **B**. LightGBM SHAP dependence plots. Note: Y-axis (per subplot): SHAP value (Shapley additive explanation value), indicating the direction and magnitude of feature influence on model output. Positive values increase prediction probability while negative values decrease it. X-axis (per subplot): Feature value (e.g., 0, 1, 2 for "Contact" feature), representing discrete or continuous measurements. Color scale: Gradient from yellow (low feature values) to purple (high feature values), reflecting actual feature magnitudes during prediction. Point distribution: Individual points represent sample-level observations in the feature-SHAP space. Interpretation: The functional relationship reveals how feature variations positively or negatively impact model decisions. For example, when "Contact" = 2, predominantly positive SHAP values indicate this value promotes target classification. CRD history refers to a history of chronic respiratory diseases; Drugs refers to medication usage during the current illness; Problem refers to the purpose of the internet consultation during this visit. **C**. LightGBM SHAP summary plot. Note: Y-axis: Features ranked by descending mean absolute SHAP value, with adjacent values quantifying average classification impact magnitude. X-axis: SHAP value (Shapley additive explanation) where negative values reduce predicted risk (left) and positive values increase risk (right). Color mapping: Gradient spectrum from yellow (low feature values) to purple (high feature values), standardized by the "Feature value" legend. Point distribution: Horizontally dispersed points represent SHAP value density across samples. For example, predominantly purple points (high values) clustered near zero SHAP for "Province" indicate minimal predictive influence at elevated feature levels. CRD history refers to a history of chronic respiratory diseases; Drugs refers to medication usage during the current illness; Problem refers to the purpose of the internet consultation during this visit. **D**. Distribution of mean decrease in accuracy and mean decrease in gini index for variables in random forest model. Note: Y-axis: Input features ranked by descending importance magnitude. Left subplot (X-axis): Mean Decrease in Accuracy (MDA), quantifying model accuracy reduction when features are permuted. Higher values indicate greater predictive importance. Right subplot (X-axis): Mean Decrease in Gini Index (MDG), measuring feature contribution to node impurity reduction during tree splits. Bar metrics: Length represents importance magnitude, with color gradient from yellow (low importance) to blue-purple (high importance) scaled by Importance Value. Annotation: Model parameters (ntree, mtry, node size) provided for reproducibility. CRD history refers to a history of chronic respiratory diseases; Drugs refers to medication usage during the current illness; Problem refers to the purpose of the internet consultation during this visit

Both the LightGBM model (Fig. 5A–C) and the RF model (Fig. 5D) show highly consistent core feature importance—CRD history and Drugs are key predictors of online consultation behavior (LightGBM |SHAP| = 0.358/0.287; RF-Mean Decrease in Accuracy (MDA) = 36.40/41.51). Contact exhibits the strongest individual decision-driving effect in LightGBM (|SHAP| = 0.489).

The two models differ in sensitivity to symptom variables: LightGBM more acutely captures the duration effects of Cough and Fatigue (SHAP dependence plots reveal linear trends), whereas RF emphasizes the contribution of CRD history to overall predictive stability (highest MDA). Notably, both model types identify Province and Nausea & Vomiting as redundant features, with importance values approaching zero.

## Discussion

During the initial phase of the COVID-19 pandemic, internet hospitals played a crucial role in meeting the healthcare needs of many individuals seeking COVID-19 consultation [12, 15], highlighting their significant potential in public health crises. Against this backdrop, within the HBM framework, this study explores key factors influencing these individuals’ decisions to use internet hospitals for follow-up consultations and employs various ML models to analyze these factors. The findings indicate that all the models performed well, with the RF and LightGBM models exhibiting superior predictive performance on the basis of comparisons of the AUROC and AUPRC.

### Statistical characteristics and the importance of data balancing

In epidemiological research, variable selection and data imbalance pose major challenges in model development. Particularly in analyzing complex behavioral determinants, ANOVA is a classical feature-selection method in medical research. It quickly identifies features with significant inter‐group differences based on statistical hypothesis testing, thereby reducing computational complexity. Boruta, which leverages the RF methodology, is particularly effective at handling non-linear and high-dimensional data. This capability accounts for its broad applicability in clinical research [26, 27]. Lasso regression, through L1 regularization, achieves feature sparsity, selects key predictors, and outputs a feature importance ranking. ASR, despite its high computational cost, exhaustively evaluates all possible feature combinations and uses cross‐validation to select the optimal subset, serving to validate the robustness of other methods’ results. Together, these four methods constitute a progressive feature‐selection framework of “rapid screening, sparsification, and comprehensive validation”.

In this study, the models achieving the highest AUPRC and AUROC values were those constructed using features selected by the Boruta algorithm (Fig. 3). Their performance did not differ significantly from that of the Full method built with the same dataset and ML algorithms, indicating that the Boruta method can streamline the feature set while preserving essential discriminative performance. These findings suggest that applying an appropriate feature selection strategy during model development can eliminate redundant variables, reduce model complexity, and provide clinical end-users with clearer insights into the key factors driving model predictions.

The results showed that the impact of resampling on model performance varied across different algorithms (Table 3; Fig. 3). Oversampling improved the performance of Boruta-RF and Boruta-LightGBM (e.g., accuracy, F1, and MCC), but had no significant effect on LR performance. This is consistent with the relatively strong parameter stability of LR in class-imbalanced settings [28]. Notably, such stability allows LR to achieve relatively favorable performance even on the original imbalanced data. In addition, SVR achieved the best MCC performance on the original imbalanced dataset. This suggests that, for certain models, conventional resampling strategies for handling class imbalance are not always a prerequisite for performance improvement. Compared with the traditional AUROC, the AUPRC is considered a more appropriate metric for summarizing predictive performance in imbalanced datasets [29, 30]. When negative samples far outnumber positive ones, a high AUROC can mask a low positive predictive value, whereas AUPRC directly reflects the model’s ability to identify the minority class and is more sensitive to false positives [30]. This study focuses on the minority of patients choosing a secondary Internet consultation, and AUPRC—by quantifying the PR balance for the minority class—more accurately captures the model’s performance on this critical behavior.

### Clinical applications of ML models

In recent years, ML has demonstrated remarkable potential in disease prediction, viral genome analysis, collective behavior decision-making, and medical imaging applications [21, 23, 31, 32]. This study found that the LightGBM and RF models demonstrated outstanding performance in predicting the probability of patients choosing Internet hospitals for follow-up consultations, The LR, CatBoost, and XGBoost models also exhibited favorable performance, whereas the predictive performance of KNN and SVM models was consistently lower than that of LightGBM and RF across various feature selection and resampling strategies (Fig. 3). As a lazy learning algorithm, KNN relies heavily on the distribution of samples within the local neighborhood, and the sparsity of minority-class samples makes their neighborhoods prone to being dominated by majority-class samples [33]. Similarly, SVM optimizes by maximizing the margin between classes, and the construction of its decision boundary inherently tends to be influenced by the distribution of the majority class [34]. As a result, it may show insufficient sensitivity in delineating the decision boundary for the minority-class event “SA” in our study. In contrast, LightGBM, through its Gradient-based One-Side Sampling mechanism, preferentially retains samples with larger gradients, thereby enabling the model to focus more on the minority class during training [35]. Moreover, both LightGBM and RF support cost-sensitive learning by allowing the specification of class weights, which explicitly adjust the loss function to enhance the recognition of minority classes [35, 36]. Medical datasets often include categorical variables (e.g., disease codes, medication categories). LightGBM’s native categorical feature splitting can directly handle these variables, thereby avoiding the dimensionality explosion caused by one-hot encoding. The development of Shapley additive explanation techniques has significantly enhanced its transparency in variable importance analysis [37, 38].

### Analyzing the association between individual characteristics in COVID-19 consultations and their healthcare-seeking behaviors based on the HBM

The COVID-19 outbreak coincided with the winter–spring peak of CRD. Patients with such underlying conditions exhibit heightened perceived severity of symptoms—cough, sore throat, and other suspected COVID-19 symptoms—which amplify their concerns about their health status. CRD history further reinforces their awareness of potential disease risk. The dual influence of acute symptoms and chronic history drives perceived severity, creating a behavioral pathway of “high perceived severity → online help-seeking,” consistent with the HBM’s premise that recognition of disease severity promotes health-seeking behavior. Cues to action were prominently activated in both models; in the RF model, Drugs emerged as the most critical external cue (highest Mean Decrease in Gini Index = 41.51), reflecting that individuals are in an ongoing treatment phase and that online follow-up offers a convenient means to continue medication management, thereby acting as an external cue to drive online consultation behavior. This discrepancy may be attributed to the prolonged duration of fatigue symptoms during COVID-19 infection and recovery [39, 40]. Fatigue serves as an internal cue, signaling that timely intervention is needed but that in-person visits are inconvenient, thereby driving individuals toward remote channels. These two cues together lower the decision threshold for online consultations, validating the HBM mechanism of “cues to action activate health behaviors.” Perceived benefits are chiefly reflected by Problem, which emerges as a core variable in both models (RF MDA = 29.83, rank 3; LightGBM core feature). The effect of perceived susceptibility is model-dependent: LightGBM identifies Contact as a core feature (positive impact), whereas its importance in RF is comparatively lower (MDA = 20.93, rank 5). This may be due to uncertainties regarding transmission routes in the early stages of the pandemic and public misconceptions about exposure risks [41]. At this stage, individuals’ judgments of “perceived susceptibility” to infection are biased, making it difficult to develop clear risk perceptions based on contact history alone and thus weakening their propensity to choose online consultation. This finding suggests that, within the HBM, perceived susceptibility requires a well-defined understanding of risk; when information is insufficient, its behavioral influence is attenuated. Differences in feature-weight distributions between the two models further illuminate the dynamic interplay of HBM constructs: RF is more sensitive to long-term health status, whereas LightGBM places greater emphasis on immediate risk signals and patient concerns. Notably, after an initial offline visit rules out infection, some patients elect online follow-up to minimize exposure risk. This behavior reflects an HBM-based trade-off between “perceived barriers” (infection risk during in-person visits) and “perceived benefits” (safety of remote care). When the perceived barrier of cross-infection outweighs the perceived benefit of face-to-face care, individuals naturally gravitate toward the lower-risk online channel. These insights extend the HBM’s explanatory power in healthcare-setting transitions, demonstrating that—beyond disease‐related beliefs—environmental risk perceptions within care contexts can significantly shape health‐seeking behaviors.

### Recommendations for optimizing internet hospital triage

The convenience, non-contact nature, and geographic flexibility of internet hospitals make them crucial options for follow-up consultations during the early stages of emerging infectious disease outbreaks. However, frequent consultations could strain healthcare resources and lead to inefficiencies. The scientific rigor of the triage mechanism directly affects the efficiency of resource allocation.

Based on the key variables identified by the RF and LightGBM models, we propose a hierarchical, HBM-informed intelligent triage scheme to close the loop between model insights and clinical practice: ①High-Risk Rapid Pathway (Perceived Susceptibility + Severity-Driven): For patients meeting all of the following criteria—positive contact history + cough or sore throat lasting > 3 days + CRD history—the system automatically issues a red alert. These cases are pinned to the top of the clinical dashboard, assigned priority to an infectious disease specialist, and simultaneously fast-tracked through the on-site nucleic acid testing channel. This approach both fulfills the HBM logic that “high perceived risk requires urgent intervention” and reduces false positives through multivariable overlap; ②Precision Specialty Matching (Perceived Benefit-Driven): Patients whose consultation purpose is medication management and who have a complex medication history are automatically triaged via natural language processing to the pharmacy clinic. The system retrieves their initial outpatient prescription data in real time and generates tailored medication recommendations. This streamlines online service delivery, maximizes perceived benefit, and enhances the efficiency of pharmaceutical consultations; ③Mental Health–Oriented Guidance (Cues to Action-Driven): For patients presenting with pronounced fatigue and who have had organic pathology excluded during their initial offline visit, the dashboard displays a “psychological concern” tag and triggers the Patient Health Questionnaire-9 screening. Positive screens are automatically routed to an online mental health service channel. By converting HBM’s “non-specific symptom cues” into actionable triggers, this mechanism uncovers and addresses patients’ latent psychosocial needs during the pandemic.

The integrated clinical dashboard design further reinforces closed-loop management: the left panel displays the patient queue sorted by HBM risk levels (red–yellow–green); the center panel presents a real-time heatmap of “Contact history – Symptoms – Consultation purposes,” visualizing interactions among HBM elements; and the right panel generates dynamic management recommendations (e.g., “Respiratory + Pharmacy co-consultation required for CRD history + medication inquiry”). After clinicians review each case, they can annotate corrections (e.g., “omitted epidemic-area travel history”), and these updates are fed back into the system for model retraining, continuously optimizing feature weights.

### Study limitations

Despite its contributions, this study has several limitations that warrant further refinement in future research. Requires further refinement in subsequent research. First, the dataset was derived from a single internet medical platform, which may limit the generalizability of the findings. Future studies should incorporate data from multiple platforms to enhance model applicability. Second, owing to the retrospective study design and ethical constraints, this study was unable to incorporate standardized psychometric instruments (e.g., the Generalized Anxiety Disorder-7 scale for anxiety, the Patient Health Questionnaire-9 for depression), limiting the depth of analysis of psychological drivers underlying features such as fatigue and consultation purpose, including the moderating effect of anxiety levels on decision-making. Finally, reliance on patient self-reported data may introduce recall bias (e.g., timing of symptom onset, chronological accuracy of medication history) and classification ambiguity. Future research will systematically refine behavior-driven models through strategies such as multi-platform data integration, embedding dynamic psychometric scales within Internet‐based clinics for longitudinal tracking, and cross-validation of key temporal variables using electronic health records.

## Conclusions

During the COVID-19 pandemic, public acceptance and trust in internet hospitals increased significantly. This study employed ML algorithms and variable selection methods to develop classification models, within the HBM framework, we translate ML–identified key variables into actionable triage protocols, elucidating the psychological logic of patients’ decisions to pursue online follow-up consultations. This delivers a comprehensive “theory–model–practice” closed-loop solution and offers Internet hospitals a reference pathway that combines theoretical rigor with practical value, thereby optimizing medical resource allocation during public health crises.

## Data Availability

All data generated and analyzed during this study are included in this published article. The raw data supporting the conclusions of this article will be made available by the authors, without undue reservation, to any qualified researcher. Data are available from the corresponding author, Tong Su, on reasonable request.

## Abbreviations

COVID-19: coronavirus disease 2019
FA: first attendance
SA: second attendance
ANOVA: analysis of variance
Lasso: least absolute shrinkage and selection operator
ML: machine learning
MCC: matthews correlation coefficient
LR: logistic regression
SVM: support vector machine
KNN: k-nearest neighbors
RF: random forest
ASR: all subsets regression
LightGBM: Light Gradient Boosting Machine
CatBoost: categorical boosting
CRD: chronic respiratory diseases
FCNN: fully connected neural network
XGBoost: extreme gradient boosting
PR: precision-recall
AUROC: area under the receiver operating characteristic curve
AUPRC: area under the precision-recall curve
